# Are Women Really Less Competitive Than Men? Career Duration in Nordic and Alpine Skiing

**DOI:** 10.3389/fsoc.2020.539766

**Published:** 2021-01-20

**Authors:** Bernd Frick, Katharina Moser

**Affiliations:** ^1^ Management Department, Paderborn University, Paderborn, Germany; ^2^ School of Business and Economics, University of Tübingen, Tübingen, Germany

**Keywords:** ski world cup, career duration, gender differences, risk preferences, self-selection, competitiveness

## Abstract

Do women shy away from competition while men compete too much? The available, mostly experimental evidence generally supports these assumptions. However, in contrast to laboratory settings, labor markets do not have random assignment of workers. Instead, individuals—professional athletes and corporate executives—self-select into specific occupations. Using data from Alpine and Nordic skiing over 52 and 37 years respectively, we show that career length of men and women is virtually identical. Thus, when adequately controlling for self-selection into a highly competitive environment, differences between men and women with respect to competitiveness completely disappear.

## Introduction

Irrespective of an already large (and still growing) body of research, neither sociology nor (behavioral) economics has yet convincingly explained the persistent gender pay gap (e.g., Kunze, 2008; Kunze, 2017) or the massive underrepresentation of women in e.g. managerial positions (e.g., Bertrand and Hallock, 2001; Frederiksen and Halliday, 2015).

Recently, gender differences in mental attitudes and dispositions—especially risk preferences and competitive orientations—have been identified as a possible reason to explain differences in the behavior of men and women. However, these differences in attitudes and dispositions (as well as in actual decision-making) have so far been primarily investigated in laboratory experiments. In her extensive review of these experiments, Niederle (2014) therefore calls for field evidence complementing the available findings. Moreover, she calls for evidence that the documented differences in psychological traits do indeed account for “a significant fraction of gender differences in economic decisions relevant to (…) market (…) outcomes of women and men,” which would, in turn, document “the external relevance of gender differences in competition (…) and risk” (Niederle, 2014, p. 8). In a recent review of the literature, Buckert et al. (2017) discuss various factors that have been found to have an impact on self-selection into tournaments: risk preferences (e.g., Niederle and Vesterlund, 2007), personality (Müller and Schwieren, 2012), and self-confidence (e.g., Niederle and Vesterlund, 2007; Croson and Gneezy, 2009).

Apart from the well-documented gender differences in risk aversion and competitive orientations, men and women have been found to differ, inter alia, in their response to feedback. While women mainly react to information on their own performance, men seem to respond more to their beliefs over the competition they will face in the future (e.g., Wozniak, 2012; Berlin and Dargnies, 2016). After having lost in a competition, the responses of men and women seem to differ again: While men tend to select more challenging tasks in the future, women lower their performance (e.g., Buser, 2016; Buser and Yuan 2019). Another important finding is that while stress (measured as salivary cortisol) increases women’s probability to enter into a tournament, this effect does not exist among men (Buser et al., 2017; Cahlíková et al. 2019). However, with increasing experience in competitive environments—such as sport—the willingness to enter in competitive systems increases for women as well as for men (Comeig et al., 2016). Not surprisingly, therefore, Filippin and Crosetto (2016) in their survey of the experimental literature and re-analyses of the available data find that gender differences in risk attitudes are largely task-specific and disappear once the idiosyncrasies of the task are adequately controlled. A similar picture emerges with respect to competitive orientations: When controlling for confidence, actual performance, beliefs about relative performance as well as the characteristics of the specific task, the gender difference completely disappears (e.g., Günther and Neslihan, 2010; Kamas and Preston, 2012; Hardies et al., 2013; Dreber et al., 2014). Moreover, Flory et al. (2018) emphasize that gender differences in competitive orientations vary across the age distribution with older women being as competitive as observationally similar men.

In this paper, we address an issue raised by Niederle and Vesterlund (2007), p. 1100 that “much may be gained if we can create environments in which high-ability women are willing to compete” in that we investigate the performance of women and men in a highly competitive environment into which both, men and women, have self-selected. Assuming that rational individuals constantly compare the (expected) costs of and the (expected) returns to the activities they engage in and that these individuals withdraw from a particular activity as soon as the marginal costs exceed the marginal returns (e.g., Coleman 1990), we contribute to the discussion on the impact of tasks on gender differences in competitive orientations in a particular way. Using field data from Alpine and Nordic (cross-country) skiing over an extended period of time (since the respective first World Cup season; 1967 in Alpine, and 1982 in Nordic skiing) until the season 2018, we identify the determinants of career length of observationally similar men and women in a particular competitive environment. In general, the careers of professional athletes appear skewed toward early exit (Witnauer et al., 2007). We interpret career length (i.e., the time between entry in and exit from the Ski World Cup) as a measure of risk preferences and competitive orientations of men and women as exit decisions that are unrelated to injuries reflect how professional athletes cope with individual success as well as failure. If women were more risk averse and/or less competitive, their careers should be shorter than those of equally (un-)successful men as they would be particularly discouraged by poor performance. Longer careers, in turn, would suggest that female athletes are less risk averse and have a stronger competitive orientation than men. Professional athletes—be they male or female—have deliberately chosen to pursue a career in a highly competitive environment with payoffs that are typical for a winner-take-all situation. Since we control for self-selection into this highly competitive environment, we do not expect to observe any differences in career duration of male and female athletes whom we thus assume not to differ with respect to risk preferences and competitive orientations. By comparing the career lengths of both genders in Alpine and Nordic skiing, we address the following research question: What are the determinants of men’s and women’s career durations when both compete under similar conditions?

Thus, the goal of our paper is to compare career length of men and women and their reactions to success and failure under virtually identical conditions. In the environment we study, men and women compete under identical rules (in the same type of competitions) and have identical returns to performance in terms of prize money^1^ as well as World Cup points^2^. Moreover, the physical requirements like e.g. slope or distance are similar (admittedly not identical) for men and women and the opportunity costs of quitting are more or less the same. Finally, professional male and female skiers represent a highly self-selected population of particularly talented and motivated individuals who are unlikely to differ in their motivations and dispositions suggesting that their response to success and failure should be more or less identical. Since we study individuals who have self-selected into a highly competitive environment, we do not expect to find statistically significant differences in the length of these athletes’ careers.

Research on (the determinants of) career length in individual professional sport is still in its infancy. Most of the existing research has so far used data from team sports. The few papers that have analyzed career length of athletes in individual sports have looked at male athletes only and none of these have used data from winter sports. To the best of our knowledge, data from FIS World Cup events have so far only been used by Feess et al. (2016) to investigate whether ability, rank and gender have an impact on risk-taking behavior in sequential tournaments and by Che and Humphreys (2013) who analyze data from FIS Alpine Skiing World Cup events to test whether women respond in a similar way to tournament incentives than men.

The remainder of the paper is organized as follows: In the next section, we summarize the literature we consider particularly relevant in our context to address the research questions outlined above. “Data and Descriptive Statistics” section includes a description of the data and provides some descriptive statistics. In “Econometric Findings” section, we present the results of our econometric analyses. “Conclusion” section summarizes our main findings and concludes with implications for management as well as future research.

## Related Literature

Before starting the review of the literature we consider relevant in our context we emphasize that we are familiar with the already large (and still growing) body of literature using lab experiments to analyze e.g. the impact of personality characteristics on risk preferences and competitive orientations among men and women and boys and girls, respectively. However, we refrain from including this literature in our review as these mostly commendable studies typically take great care in randomly allocating participants to the various treatments, while failing to recognize that the labor market is hardly random (for reviews of the literature see e.g., Croson and Gneezy, 2009; Niederle and Vesterlund, 2011 as well as Dechenaux et al., 2015). Summarizing the available—mostly experimental—evidence, Croson and Gneezy (2009), p. 464 concluded that “women are more reluctant than men to engage in competitive interactions like tournaments, bargaining, and auctions. Additionally, men’s performance, relative to women’s, is improved under competition. Thus, as the competitiveness of an environment increases, the performance and participation of men increase relative to that of women.” The females’ reluctance is usually explained by their more pronounced risk aversion. However, most experimental studies seem to suggest that the observable pattern of gender differences in competitiveness is associated with excess entry of men due to overconfidence rather than due to differences in risk aversion. Moreover, the available evidence seems to suggest that women “choke under pressure” more than men do (Niederle and Vesterlund, 2009, p. 9). Finally, some evidence—again mainly experimental—emphasizes the importance of the socio-cultural context as a factor influencing competitive orientations of men and women.

While the laboratory experiments are carefully designed, they have difficulties in distinguishing between the most likely sources of gender differences in competitiveness—the incentive effects of tournaments on the one hand and self-selection of persons with a “competitive motivation” on the other hand. Professional athletes—similar to top managers—have self-selected into activities for which they are particularly talented, qualified and motivated. Thus, the literature we consider in this section comes from two different strands of research: First, papers analyzing gender differences in performance due to *self-selection*, *pressure, choice of strategy* as well as *response to incentives* and, second, studies looking at *career length* in professional sports. With respect to the latter, little to nothing is available that compares the behavior of men and women. For our literature review, we have deliberately chosen examples from a wide range of sports that not only differ in terms of the prize money available to the top performers but also with respect to their rules and regulations, their physical requirements, and the public interest they receive. If we find the responses of men and women to be similar across different sports we can be more confident that their behavior is not driven by the idiosyncrasies of a particular sport.

### Gender Differences in Self-Selection in Professional Sport^3^

Until the 1960s, far more men than women chose to become professional athletes in the Western world, i.e. self-selected into a highly competitive environment. However, in the last 50 years this gender gap in self-selection has decreased considerably due to the gradual erosion of social barriers allowing women to become professional athletes and to train as hard as comparably talented men.^4^ More recently, a similar development has occurred in many African and Asian countries with the consequence that today in e.g., track and field as well as in road running athletes from East African countries dominate among men as well as women (women were not allowed to participate in marathons before 1973). This is not surprising as the incentives to train properly nowadays are similar for male and female athletes because the “returns to winning” are identical since the early 1980s.

Using data from ultramarathons, Frick (2011b) found that initially, that is around the year 2005, the pool of female runners was far more heterogeneous than the pool of male runners. However, over time the performance differential between men and women declined rapidly. Particularly interesting in this context is that this decline is much larger on the 100 km, that is, the race where incentives to perform well are particularly high (due to, e.g., the existence of a World Cup). Thus, the presence of monetary as well as non-monetary incentives induced a gradual process of self-selection into professional athletics, slowly leading to the emergence of a competitive field. Since the returns to self-selection are considerably higher on the 100 km than on the 50 km, the performance differential between the top male and female athletes on the former distance is far smaller than on the latter and increases significantly more on the 50 km distance as one moves up higher in the ranking.

In a companion paper using longitudinal data from middle- and long-distance foot races Frick (2011a) revealed that the rank corrected percentage difference in finishing times between male and female athletes is particularly small on the 3,000 m outdoor track, the 5,000 m outdoor track, the 10,000 m outdoor track and the marathon. These track races are equally attractive to men and women because they are part of the International Association of Athletics Federation's (IAAF) championships and/or the annual “Golden League” meetings (the most lucrative events in track and field athletics apart from the city marathons). Marathons, in turn, are also equally attractive to male and female athletes as the prize purses have increased considerably in the recent past and are now equal for men and women. Thus, it appears that in the race types where prize money and/or prestige is particularly high, the gender difference in competitiveness is significantly lower than in races that are—for various reasons—considered less attractive. Frick (2011a) also found that the rank corrected time difference between male and female athletes has declined considerably over the last 40 years and is narrowing by about 2% per year. This suggests that women are catching up rapidly and that the gender gap in competitiveness that has been documented in most previous studies will have disappeared soon in this (admittedly idiosyncratic) context. Thus, social change and economic incentives induce similar numbers of men and women to self-select into athletic careers.

Potential differences in self-selection across genders were analyzed by Nekby and Vahtrik (2008), who used data from the 2005 and 2006 editions of the “Midnight Race” held in Stockholm every year in June. In 2005, runners were allocated into start groups based on their previous or expected performance by the race organizers. In 2006, runners were given the opportunity to self-select into a start group based on their individual assessment. The authors found that women who self-select into a start group with faster runners tend to be over-confident, i.e., their finish time is slower than expected. Using data from seven editions of the “Santa Barbara State Street Mile” in the years 2002–2008, Garratt (2013) found that not only women, but also older men try to avoid competitive pressures by self-selecting into slower start groups, suggesting that over-confidence is particularly widespread among younger men.

### Gender Differences in Performance “Under Pressure”

An already large number of studies using data from different individual sports have analyzed the behavior of male and female professional as well as recreational athletes during competition, i.e., *under pressure*. Using data on expert chess players, Gränsmark (2012) found that women perform worse under time pressure, i.e., when the amount of time available for the next move decreases as time progresses. The quality of the moves of women is better in the first half of the game, whereas men’s performance is better in the second half.

Using data from high jump and pole vault competitions, Böheim and Lackner (2015) found that although competitive pressure induces men and women to take more risky decisions (i.e., to skip attempts and move to the next height), this change in behavior is more pronounced among men than among women.

Using data from the New York City Marathon events in the years 2007 thru 2014, Birk et al. (2016) showed that when elite-female athletes are exposed to the presence of men, their performance is negatively affected with the effect being larger among lower-ability runners. More precisely, the pace of a female elite runner decreases by about 0.9% when being overtaken by men, a finding that the authors attribute to the “psychological effects” of competition. Booth and Yamamura (2016) used a sample of more than 400,000 person-race-observations from Japanese speedboat racing in the period April 2014 to October 2015 and found that in gender-mixed races, women’s performance is slower than in women-only races. Men, however, are faster in gender-mixed races compared to men-only races. Moreover, men behave more aggressively in gender-mixed races. Women, in contrast, are less aggressive in gender-mixed races than in women-only races. Aggressive behavior can be considered a choice variable in contests as well as in everyday life. Therefore, the next section focuses on (possible) differences in choice of strategy among men and women.

### Gender Differences in the Choice of Strategy

Recently, a number of studies using large samples on expert chess players have documented statistically significant differences between men and women in terms of choice of strategy: First, Gerdes and Gränsmark (2010) found that men choose more aggressive opening strategies when playing against women. Women, in turn, choose aggressive strategies only when playing against better-ranked women. Second, Dreber et al. (2013) found that men choose significantly riskier strategies when playing against a physically attractive female opponent although this choice of strategy increases the probability of losing the match. Similar differences in strategy choice between men and women have been found in professional tennis. Using data from Grand Slam Tournaments, Paserman (2010) found that women commit significantly more unforced errors at crucial junctures of the match. Moreover, men and women choose a more conservative and less aggressive style of play as points become more important (as measured by serve speed, first serve percentages and rally length), but this change in behavior is more pronounced among women. Analyzing gender differences in line-call challenges in major tennis tournaments all over the world, Anbarci et al. (2016) found that male players’ challenges are more likely to be a response to their opponents’ behavior. At tiebreaks, women display a higher probability than men to reverse an umpire’s unfavorable call. Men, however, make relatively more unsuccessful challenges. Finally, Jetter and Walker (2015) provided strong evidence for corrupt behavior on the men's tour, where “bubble players” are found to be substantially more likely to beat better ranked opponents when a win is desperately needed. No such evidence was found on the women's tour, suggesting considerable gender differences with respect to corrupt and unethical as well as collusive behavior.

### Gender Differences in the Response to Incentives

Apart from gender differences in performance under pressure and in the choice of strategy, differences between men and women can also be observed in the response to *incentives*. Gilsdorf and Sukhatme (2013) used data from the 2009 PGA and LPGA Tour and found that, when looking at total scores, men and women respond similarly to (changes in) incentives. However, looking specifically at final round scores they find that female golfers respond more strongly to incentives in the sense of improving their performance than male golfers. Leeds et al. (2013), in turn, used data from figure skating contests in the 2009–2010 season and also found that women respond more strongly to incentives than men. Moreover, female figure skaters display a more resilient behavior when confronted with negative feedback and do not avoid competition with other elite skaters.

### Career Length in Professional Sport

Much of the existing literature on career length in professional sports focuses on *team sports*. Atkinson and Tschirhart (1986) analyzed career length data on 260 National Football League (NFL) players active from 1971 to 1980 using hazard models. The average career length in this population was 4.5 seasons. First year NFL players experienced increasing hazard early in their career but players who survived this early career “shakeout” experienced a falling hazard rate for the remainder of their careers. Team and individual performance had a positive effect on career length. Staw and Ha (1995) investigated career length data for NBA players picked in the first two rounds of the drafts held from 1980 through 1986. 275 players from this population played at least one season in the NBA of whom 184 had exited by the 1990–91 season. The average career length was just under 8 seasons in this population. Results suggested that scoring performance and the total number of rebounds and blocks were associated with longer career lengths, and draft position and the number of times a player was traded were associated with shorter career lengths. Frick et al. (2007) and Frick et al. (2009) analyzed career length data for 4,116 players who appeared in at least one match in the top league of professional soccer in Germany, the Bundesliga, over the 1963–1964 to 2002–2003 seasons. The average career length was less than 4 seasons in this population. Results suggested that defenders, midfielders and forwards had shorter careers and goalkeepers longer careers. The total number of games played and goals scored per season were associated with longer careers and yellow and red cards per season had no impact on career length.

Few studies have analyzed career length in *individual professional sports*. Two of these focused on professional tennis. Geyer (2010) analyzed career length data for 614 male professional tennis players who participated in Grand Slam tournaments, Association of Tennis Professionals (ATP) tournaments, and International Series tournaments over the 1985 through 2007 seasons. The average career length in this population was 6 seasons. Player performance, in terms of tournaments won and percentage of games won, were associated with longer career lengths while lower world ranking was associated with shorter careers. Frick and Scheel (2016) analyzed career length data for 698 male professional ski jumpers competing in more than 750 different tournaments over the 1979–1980 thru 2010–11 seasons. The average career length in this population was just 4 seasons. Athletic performance, in terms of World Cup points accumulated over a season and winning World Championships, was strongly associated with longer career lengths. In addition, Frick et al. (2015) analyzed career duration in American stock car racing and in professional golf using information on career length and performance of 740 male professional automobile racers from NASCAR between 1975 and 2010 as well as 1,639 male professional golfers from the 1980/81 to 2012/13 season who played on the PGA Tour for at least one year. They find that career length was significantly different with nearly 7 seasons for the latter and less than 5 seasons for the former, suggesting that differences in physical requirements may also have an impact on the duration of an athlete’s career.

To the best of our knowledge, only one study exists so far that compares career length of men and women in professional sport. Coate and Robbins (2001) analyzed career length data for 236 male and 216 female tennis players who were ranked in the world top 50 in singles at least once between 1979 and 1994. The average career length was about 9 seasons in this population. The results suggest that there was no difference between the career length of males and females, despite significantly lower prizes for women. No performance measure was included in the empirical models. This study, however, suffers from a major problem: The physical requirements for male and female tennis players are significantly different as women play up to three sets to determine the winner of a match while men play up to five sets. We avoid this problem by comparing career length of men and women competing under similar, yet not completely identical conditions in Nordic and in Alpine skiing, to determine the winner of a tournament to rule out potential biases that may result from differences in physical requirements, levels of fatigue, injury risks, etc.

### 

 The determinants of individual career length building on the influential work by Coleman (1990), we assume rational actors with resources linked by interests and control relations. Interests, i.e. the motive governing actions, reflect a resource’s impact on an actor’s well-being while control refers to the rights to direct the use of resources. Moreover, actors are embedded in a specific social structure, i.e., interdependencies, networks, organizations and authority structures that limit them in pursuing their interests (Marsden 2005). Rational individuals constantly compare the (expected) costs of and the (expected) returns to the activities they engage in. As soon as the marginal costs exceed the marginal returns, the individual withdraws. Thus, the utility functions of competitive and non-competitive individuals are different in the sense that their appraisal of costs and benefits of a particular activity differs considerably.

In general, career duration in professional sport can be analyzed from several theoretical perspectives. Participating in professional sport over a period of time can be viewed as a labor supply decision, and analyzed in the context of standard dynamic lifecycle labor supply decisions (Mitchell and Fields, 1984). In this context, the end of the athlete’s playing career represents a decision to retire from the sport. Retirement is typically a voluntary decision made by employees based on their current and expected earnings and other factors like the value of leisure time and life expectancy. The annual earnings of professional athletes can be large, and some participants in professional sport may view their employment as a way of earning large sums in a short period of time in order to retire early.

Alternatively, the end of an athlete’s career can be viewed as a dismissal from the sport. In dynamic models of employee dismissal, inefficient employees are systematically eliminated from employment (Flinn, 1997). This approach to employee dismissals is related to labor market search models and employee-firm matching (Mortensen, 1978). The end of a career is involuntary in this case, and represents a profit maximizing decision on the part of the employer based on the contribution of the employee and the availability of alternative employees. A spell of employment ending with a dismissal can also be interpreted as an outcome of a promotion tournament (Szymanski, 2003). The organizers of professional sports contests want to attract the most talented athletes in order to maximize profits; when an athlete becomes less productive due to age or injury, contest organizers will replace that athlete with a more productive competitor, leading to an end of the spell of employment.

A spell of employment in a professional sport can also be viewed from the perspective of occupational tenure (Klee, 2013). This approach emphasizes the idea that a career in professional sports is one of several occupations that professional athletes might pursue, and focuses on matching between employer and employee and the role played by occupation-specific human capital. The end of a professional sports career may or may not be voluntary in this context, but the occupational tenure approach emphasizes the importance of other related occupations, like coaching, scouting, or providing media commentary on events, when an athlete’s human capital might be useful, as well as the earnings in these related, and perhaps other unrelated occupations.

All of these models imply that current performance, expected future performance, the presence of other employees that can perform the same job, and the value of leisure time affect the exit from employment. These models differ in whether or not the quit is voluntary, or forced on the employee by the employer. In practice, econometricians have limited information about why a professional athlete’s career comes to an end. It could be voluntary, as explained by lifecycle labor supply models, or involuntary, as explained by models of employee dismissal and tournament theory models. Models of occupational tenure can include both voluntary and involuntary career terminations. In addition, athletes may experience career-ending injuries that may or may not be observable to the econometrician. These factors make a complete understanding of the reason for the end of the employment spell difficult to determine, and also make it difficult to determine which model to apply to the econometric analysis of career duration in professional sport.

Based on the notion of rational utility-maximizing individuals and of career length as a plausible measure of risk preferences and competitive orientations we derive (and subsequently test) the following hypotheses:

**H_1_:** *Career length of male and female professional athletes is not significantly different in neither Alpine nor in Nordic Skiing*.

As we want to identify the determinants of individual career length, we analyze the impact of various measures of absolute and relative performance. Since the most obvious measure of individual performance is the number of World Cup points an athlete accumulates over the course of a season, our second hypothesis is as follows:

**H_2_:** *The more World Cup points an athlete accumulates during a season, the longer his/her career will last in both, Alpine and Nordic skiing*.

Further, since each national federation is guaranteed a limited number of starting slots only, each athlete’s performance is benchmarked against that of his/her compatriots. This suggests that the same number of World Cup points is of less value for a member of a strong nation (such as Austria in Alpine skiing or Sweden in Nordic skiing) than for a member of a weak nation (such as, e.g., Germany in Alpine or France in Nordic skiing). Thus, we hypothesize:

**H**_**3**_ **:** *The higher the number of competitors within a national federation, the shorter the careers of both, male and female athletes in Alpine and Nordic skiing*.

In some seasons, a small number of athletes dominate most of the contests and accumulate disproportionately large shares of World Cup points, leading to disappointment and frustration among the remaining athletes. Therefore, we formulate our fourth hypothesis as follows:

**H**_**4**_ **:** *A higher concentration of World Cup points leads to shorter careers of male and female athletes in Alpine and Nordic skiing*.

An athlete who performs well compared to his/her compatriots in the sense that s/he assembles a large fraction of World Cup points for his/her national federation is likely to survive longer. Thus, our fifth hypothesis reads:

**H**_**5**_ **:** *A higher percentage of World Cup points leads to longer careers of male and female athletes in Alpine and Nordic skiing*.

Finally, since prize money (as well as the monetary value of endorsement contracts, the content of which remains usually private and confidential) has increased over time, the opportunity costs of quitting have also increased. Consequently, our sixth hypothesis is as follows:

**H**_**6**_ **:** *Over time career length increases for both, men and women, in Alpine as well as in Nordic skiing*.^5^

## Data and Descriptive Statistics

Our approach is similar to the one adopted by the studies on exit discrimination in professional team sports (e.g., Hoang and Rascher, 1999) in the sense that we compare career length of male and female athletes in two so far completely neglected winter sports holding individual productivity constant. However, in our study we use career length as a measure of risk preference and competitive orientations.

We collected data on career length and performance of male and female Alpine as well as cross-country skiers from the websites of “Fédération Internationale de Ski” (FIS) for all seasons since the implementation of that particular sport’s World Cup (1967 for the former and 1982 for the latter). The data is available at https://data.fis-ski.com/cross-country/cup-standings.html and https://data.fis-ski.com/Alpine-skiing/cup-standings.html. Our final data set includes each individual that ever won at least one World Cup point over the period 1967 (1982) thru 2018, yielding a data set with 4,151 individuals (1,835 women and 2,316 men) and 18,776 athlete-year-observations.

It appears from Table 1 above that average career length of men and women in both sports is between 4 and 5 years and differs only slightly (by 0.3 years in Alpine and 0.1 years in Nordic skiing). Moreover, career interruptions—which may be due to injuries, degradation to the regional cup competitions (such as the European Cup) or doping bans—are rather frequent events for both, men and women (see Table 2). Contrary to expectations, very few of the female athletes interrupt their career due to pregnancy (it may well be that female athletes exit from professional skiing when they learn that they are pregnant). Athletes that have tested positively predominantly come from countries of the former Soviet Union and very often return to the World Cup after two years, suggesting that their second best alternative is unemployment.

**TABLE 1:** Descriptive statistics I.

Genderand sport	Individuals	Observations	Exits	Average career length^a^
Men, Nordic	1,108	4,404	953	3.97
Women, Nordic	804	3,274	696	4.07
Men, Alpine	1,208	6,136	1,048	5.08
Women, Alpine	1,031	4,962	896	4.81
Total	4,151	18,776	3,593	4.48

^a^ Column 3/Column 2

**TABLE 2:** Descriptive statistics II.

Gender and sport	Individuals with gap	Percent of individuals^a^	Time on gap (Years)	Average time on gap^b^
Men, Nordic	286	25.8	403	1.41
Women, Nordic	237	29.5	299	1.26
Men, Alpine	391	32.4	478	1.22
Women, Alpine	275	26.7	306	1.11
Total	1189	28.6	1,486	1.25

^a^ (Column 2/Column 2, Table 1) *100.

^b^ Column 4/Column 2.

**FIGURE 1:**
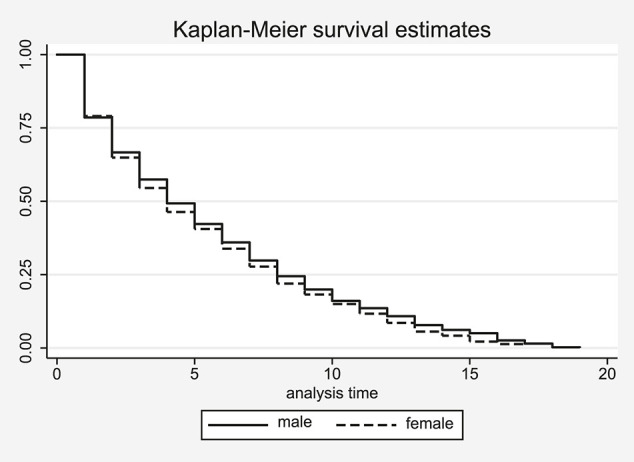
Kaplan–Meier estimation of career length in Alpine skiing by gender.

**FIGURE 2:**
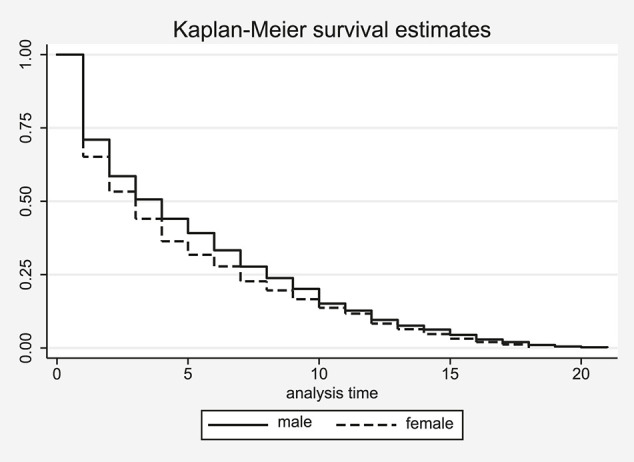
Kaplan–Meier estimation of career length in Nordic skiing by gender.

The Kaplan-Meier survival estimates seem to suggest that the conditional probability of women to exit is higher than the respective probability of men (Figure 1 for Alpine and Figure 2 for Nordic skiing). This impression, however, is not supported by the results of the log rank test for equality of the respective survivor functions as the test statistics fail to reach conventional levels of statistical significance in both cases, suggesting that men do not survive longer in their sport.

Note that the distribution of the survivor functions in both sports is similar in the first year of male and female athletes’ careers in Alpine, but not in Nordic skiing. About 20% of male as well as female athletes in the former sport have a career of only one season, shown as the first step down at the left hand side of Figure 1. After the first season, the career lengths begin to differ between male and female athletes with the probability for male athletes to remain on the tour always being slightly higher than for women. However, the difference in the survival probabilities never exceeds two percentage points. For Nordic skiing the picture is completely different (Figure 2). Here about 30% of the male but 40% of the female athletes exit the tour after their first season already. This difference in the survival probabilities continues to grow for the next four seasons before decreasing again after the fifth year on the tour.

## Econometric Findings

While this unconditional, non-parametric analysis of career length uncovers some interesting patterns, most research on career length performs conditional, parametric or semi-parametric analyses of the performance of athletes to better understand the determinants of career length. Parametric and semi-parametric methods can condition observed career length on other observable factors like performance that are known to affect career length systematically in other settings. In this conditional analysis of career length, we use two different data sets—one for each sport—because we assume that the baseline hazard function varies systematically across sports and we therefore need to estimate sport-specific models.

First, we explain observed career length using covariates that reflect the performance of the athletes because the previous literature on career length in sport surveyed above finds performance to be closely linked to career length theoretically and empirically. The performance measure is the annual number of World Cup points assembled by each Alpine or Nordic skier. Since this system is the same for men and women, we only have to control for changes in that system that have occurred over time. We can do this by either including in the estimations a vector of dummy variables representing the different “point regimes” or by standardizing the World Cup points across the different regimes of both disciplines and both genders (mean = zero; standard deviation = 1).

Second, we control in our estimations for an athlete’s individual performance compared to his/her compatriots as well as relative to their immediate competitors in the World Cup tournaments. Since each national federation is guaranteed a limited number of slots only, athletes from strong federations (e.g., Austria in Alpine and Norway in Nordic skiing) face a substantially higher risk of exit (or relegation) than observationally similar athletes from weak federations (e.g., Germany). Thus, an athlete’s career length is not only affected by his/her individual performance but also by the performance of his/her compatriots. Strong athletes from nations with a particular tradition in Alpine or Nordic skiing are, therefore, always threatened by relegation while even rather weak athletes from weak nations may be able to survive in their sport for quite a while. We measure an athlete’s performance relative to his/her compatriots by the percentage of World Cup points accumulated by athlete i in season j for national association k, reflecting the different nations’ level of competitiveness.^6^

Third, the higher the number of competitors, the lower an individual’s success probability and the more a sport is dominated by a single athlete winning most of the contests, the shorter will—on average—be the remaining athletes’ careers. To control for these effects, we include in our estimations the number of athletes winning World Cup points in a particular season as well as the concentration of World Cup points, measured by the Gini coefficient calculated separately year by year for men and women in each of the two sports.

Fourth, to proxy for the increase in prize money and in (presumably lucrative) endorsement contracts (on which only anecdotal evidence is available) we include in our estimation a linear time trend.

Finally, we interact each of our explanatory variables (World Cup points, percentage of World Cup points assembled by a particular athlete for his/her national association, number of competitors winning at least one World Cup point, concentration of World Cup points and time trend) with a dummy for gender to check whether women respond in a similar or different way than men to changes in competitive pressures. Table 3 displays the descriptive statistics.

**TABLE 3:** Descriptive statistics for Alpine and Nordic skiing.

Variables	Mean	Std. Dev	Min	Max
Alpine skiing (*n* = 11,098 observations)				
Female (1 = yes)	0.447	—	0	1
Standardized world cup points (z_wcp)	0.000	0.995	−0.976	7.175
Number of competitors (competitors)	118	31	35	161
Concentration of world cup points (concentration)	0.615	0.031	0.493	0.668
Percentage of world cup points (perc_wcp)	0.074	0.174	0.000	1.000
Time trend (trend)	31	14	1	52
Nordic skiing (*n* = 7,678 observations)				
Female (1 = yes)	0.426	—	0	1
Standardized world cup points (z_wcp)	0.000	0.995	−1.051	7.387
Number of competitors (competitors)	119	41	35	186
Concentration of world cup points (concentration)	0.646	0.056	0.475	0.735
Percentage of world cup points (perc_wcp)	0.059	0.149	0.000	1.000
Time trend (trend)	22	10	1	37

The Cox proportional hazard model is the standard econometric method used to analyze duration data containing right-censored and time-dependent observations. This semi-parametric model has two advantages over other proportional hazard models: First, it can be applied to right-censored data, which is important here since about 10% of the skiers analyzed are still active at the end of the sample period. For these competitors, their exit from the competition has not yet occurred, leading to right-censored observations. Second, the Cox model makes no assumptions about the underlying survival distribution and can incorporate time-dependent variables and also deal with left-truncation. Left truncation occurs when athletes in the sample have careers that began before the start of the sample period (i.e. before 1967 in Alpine and 1982 in Nordic skiing).

In addition to a Cox hazard model, we estimate a parametric hazard model. Parametric hazard models exploit the information about career length differently than Cox hazard models, and can be interpreted as regression models (Cleves et al., 2008). The specific form of parametric hazard models depends on the distribution of the dependent variable, career length. In this setting, the Akaike Information Criterion (Akaike, 1974) indicates that in the case of Nordic skiing a Weibull model and in the case of Alpine skiing a Gamma model is preferable to other parametric models like the log-logistic model.

### 

 Our most important findings are as follows first, career length of women in both, Alpine and Nordic skiing exceeds career length of men, i.e., female athletes exit later than observationally similar male athletes (the respective coefficient in the Cox model is negative). For Alpine skiing this is further supported by the significantly positive coefficient in the Gamma model. For Nordic skiing, however, the respective coefficient turned out to be insignificant in the Weibull model. These findings contradict H_1_ and suggest that women are definitely not less competitive than men—when adequately controlling for self-selection into a highly competitive environment.

Second, World Cup points have the expected statistically significant and positive impact on career length in both sports in all four models, supporting H_2_: Other things equal, each additional World Cup points reduces the probability of exit. However, the statistically significant coefficient of the respective interaction term suggests that additional World Cup points reduce the exit probability of women less than they reduce the exit probability of men in Alpine skiing, while in Nordic skiing the coefficients of the interaction terms are not significant.

Third, the number of contestants each athlete competes against is not statistically significant, suggesting that it does not influence the career length in neither Alpine nor Nordic skiing in general and, therefore, contradicting H_3_. In Nordic skiing, however, it is statistically significant for women, meaning that they have a higher risk of exiting if the number of competitors increases. This latter finding suggest that poorly performing women in Nordic skiing are replaced earlier by upcoming athletes than poorly performing men.

Fourth, an increase in the concentration of World Cup points is not statistically significant for Alpine, but for Nordic skiing, as it reduces the probability to exit. However, the interaction effect is statistically significant in both, Alpine and Nordic skiing, suggesting that women’s careers are shorter in case that few athletes dominate during a season, which we interpret as partial support of H_4_.

Fifth, neither the coefficient of the percentage of World Cup points nor the interaction with the female dummy are statistically significant, implying that the share of World Cup points an athlete has assembled for his/her national federation in the previous season does not influence the probability to exit, neither in Nordic nor in Alpine skiing. These findings are incompatible with H_5_.

Sixth, over time career duration increases in Alpine, but not in Nordic skiing (the coefficient of the linear time trend is statistically significant in all models, but is negative in the Cox model in Alpine and positive in Nordic skiing, while in the Gamma and Weibull models they are both positive). Thus, in Nordic skiing career length decreases. Since the coefficient of the interaction term is statistically significant in Nordic skiing only, women’s career length has increased in this sport over time, partly supporting H_6_.

## Conclusion

Summarizing, our findings suggest that women in general have longer careers than men in both, Alpine and Nordic skiing. However, the length of their careers is slightly lower than that of comparable men in case they are particularly successful (i.e. have assembled more World Cup points in Alpine skiing) or in case they have more competitors (in Nordic skiing). Moreover, the negative career length effect of a high points concentration is statistically significant only for women (both, in Alpine and Nordic skiing). Thus, we have to partially reject our second hypothesis as the returns to World Cup points in terms of career duration are lower for women than for men and we also find only partial support for our third hypothesis as career length is negatively affected by the number of competitors only in the case of female athletes. Further, we find only partial support for our fourth hypothesis as a higher concentration of World Cup points only affects career length of women, but not of men. As the percentage of World Cup points does not have any significant effect, we also have to reject our fifth hypothesis. Finally, since over time, career duration is increasing only for women in Nordic skiing, we find again partial support for our sixth hypothesis.

Apart from these minor differences, it appears that once self-selection is taken into account, women are at least as competitive as men, i.e. career duration in a highly competitive environment is independent of gender. Thus, we find support for our first hypothesis, stating that there is no systematic difference in career length of men and women, neither in Alpine nor in Nordic skiing. This suggests that men and women are equally good in coping with success as well as failure. What is perhaps surprising is the fact that we do not observe shorter careers among less successful women, suggesting that these women are not discouraged faster than equally unsuccessful men. On the contrary, all we find is that the top female athletes have shorter careers in both Nordic and Alpine skiing, suggesting the availability of (financially) lucrative alternatives (as e.g. TV commentators and/or models). With regard to our research question, we find that an individual’s performance is the most important determinant of career length. The number of competitors as well as the concentration of sporting success has only a small, yet in some specifications statistically significant, effect on individual career length.

Our findings seem to contradict the results reported by Cunningham et al. (2019), who found that women have significantly shorter careers as assistant or head coaches in the NCAA. They argue that occupational constraints seem to limit women’s aspirations and intentions to remain in coaching. Our results, however, suggest another explanation: As long as women compete with other women (in e.g. Alpine and Nordic skiing), they are as competitive as men. If, however, they have to compete with men (be it for coaching or for top management positions in sport), they withdraw (more or less) voluntarily, as they do not want to compete with men.^7^

Our findings are not completely consistent when looking at the impact of competitive pressure on career length across the two different sports. Why are women’s careers adversely affected by the presence of more competitors in Nordic, but not in Alpine skiing? Why are women’s careers getting shorter over time in Nordic, but not in Alpine skiing? These questions clearly need to be addressed in future research.

Thus, the results of previous studies finding significant differences between men and women with respect to competitive orientations are likely to be biased due to inadequate controls for self-selection. One of the few exceptions in this context is Azmat and Ferrer (2015) who implicitly control for self-selection into a particularly competitive environment by comparing the performance of young female and male lawyers. They find large gender differences in productivity and argue that these are due to significant differences in weekly working hours rather than differences in these individuals’ mental dispositions and attitudes. Another interesting approach adequately addressing the issue of self-selection is Apicella et al. (2017) who fail to find any statistically significant difference between men and women when competing against their own previous performance instead of competing against other people, which is mostly in line with our findings reported above.

From the point of view of rational choice theory, or results are as expected: Self-selection into occupations is not random, and some occupations are chosen by persons who have an inclination toward non-monetary rewards (Lazear 2018, p. 209). One example is professional sport such as Nordic and Alpine skiing requiring a lot of talent, implying significant hardship and low average compensation. Those who choose occupations like these value social rewards (like e.g. the thrill of competition and enjoying a particular lifestyle) over monetary ones. This is apparently equally valid for men and women.

The managerial implications of our findings are straightforward: Controlling for self-selection is crucial when analyzing career duration of men and women particularly in highly competitive environments. Hence, we would expect not to find statistically significant differences in career length of male and female consultants, investment bankers or lawyers, although in all these jobs women continue to be massively underrepresented. However, women who self-select into one of these occupations are likely to have similar, if not identical aspirations, motivations, and mental dispositions than observationally similar men. In order to encourage qualified women not to shy away from competition, firms should organize e.g. promotion tournaments in a way that women compete with women and men with men.

**TABLE 4:** The impact of gender on career length in Nordic and Alpine skiing.

	Alpine skiing		Nordic skiing	
	Model 1.1 Cox	Model 1.2 Gamma	Model 2.1 Cox	Model 2.2 Weibull
Female	−2.166*	1.388*	−1.656*	−1.123
	(0.961)	(0.589)	(0.705)	(0.712)
z_wcp	−1.528**	1.044**	−1.159**	−1.448**
	(0.090)	(0.056)	(0.089)	(0.095)
zwcp_female	0.384**	−0.223**	0.065	0.052
	(0.118)	(0.076)	(0.133)	(0.144)
Competitors	0.002	−0.001	−0.000	−0.001
	(0.002)	(0.001)	(0.002)	(0.002)
comp_female	0.002	−0.002	0.012**	0.017**
	(0.003)	(0.002)	(0.004)	(0.004)
Concentration	0.844	−1.057	−6.594**	−6.089**
	(1.177)	(0.716)	(1.004)	(1.007)
conc_female	3.914*	−2.425*	5.250**	4.535**
	(1.649)	(1.019)	(1.427)	(1.426)
percent_wcp	0.133	−0.118	−0.250	−0.191
	(0.161)	(0.096)	(0.257)	(0.264)
percent_wcp_female	−0.157	0.136	−0.187	−0.342
	(0.260)	(0.157)	(0.417)	(0.428)
Trend	−0.031**	0.021**	0.040**	0.038**
	(0.005)	(0.003)	(0.008)	(0.008)
trend_female	−0.002	0.003	−0.075**	−0.090**
	(0.007)	(0.005)	(0.011)	(0.011)
Constant	—	2.255**	—	−0.063
		(0.409)		(0.500)
Lnsigma	—	−0.5071734**	—	—
		(0.021)		
Kappa	—	1.012953**	—	—
		(0.061)		
ln_p	—	—	—	0.3269195**
				(0.174)
LL Null model	−13,337.57	−2,842.983	−11,070.08	−2,561.685
LL Full model	−12,845.37	−2,227.55	−10,783.68	−2,128.746
N of observations	11,098	7,678		
N of individuals	2,239	1,912		
N of exits	1,944	1,649		

Robust standard errors (clustered at athlete id) in parentheses.

*p < 0.05, **p < 0.01.

## Data Availability Statement

Publicly available datasets were analyzed in this study. This data can be found here: www.fis-ski.com.

## Author Contributions

All authors listed have made a substantial, direct, and intellectual contribution to the work and approved it for publication.

## Conflict of Interest

The authors declare that the research was conducted in the absence of any commercial or financial relationships that could be construed as a potential conflict of interest.

## Footnotes

1 In the 2016–17 season, the prize money in Alpine skiing was 110,000 CHF per race of which 45,000 CHF went to the winner (some races have slightly lower/higher purses). The number of races was identical for men and women (at 37) as was the prize money (see Supplementary Table S1 for a list of the top earners in Alpine and Nordic skiing).

2 Although there are considerable differences in the amount of prize money that is allocated among the athletes, Alpine (including slalom, giant slalom, super G, downhill and combined) and Nordic skiing (sprint as well as endurance cross-country events on various distances) use the same distribution of prize money and World Cup points. The current scoring system has been used since the 1991–92 season (see Supplementary Table S2 for the distribution of World Cup points and prize money).

3 Since we concentrate on findings from professional sport, we exclude here the “running studies” based on field experiments mostly in elementary schools by e.g., Gneezy et al. (2003), Gneezy and Rustichini (2004), Dreber et al. (2011), Cardenas et al. (2012), Khachatryan et al. (2015) as well as Sutter and Glätzle-Rützler (2015).

4 While at the 1896 Summer Olympics in Athens, women were not allowed to participate, the percentage share of female competitors at the 1900 Summer Olympics in Paris was 2.2 percent. In 2012 (London) and 2016 (Rio de Janeiro), the respective shares had increased to 44.3 and 45.0%, respectively. In the Winter Olympics the share of female participants increased from 5.0% (Chamonix 1924) and 5.8% (St. Moritz 1928) to 40.3% (Sotschi 2014) and 41.5% (Pyeonchang 2018).

5 Apart from the increase in prize money and endorsement contract revenues, other factors such as improvements in training and conditioning methods, advances in the treatment of injuries and in diet may play an important role too. These effects, however, are difficult, if not impossible to separate from each other.

6 Hence, similar to, for example, the U.S. and Jamaican sprint trials or the domestic qualifying events for marathon runners in Ethiopia and Kenya, a nomination for the Austrian Alpine or the Norwegian Nordic World Cup team is presumably more difficult to achieve than a spot among the Top 30 in a given World Cup competition.

7 Our interpretation is consistent with the findings reported by Wicker and George (2019), who found the dismissal probability of soccer head coaches following poor team performance to be the same for men and women.
