# Supplementary material for: Are Women Really Less Competitive Than Men? Career Duration in Nordic and Alpine Skiing
Source: Front Sociol. 2021 Jan 20;5:539766. doi: 10.3389/fsoc.2020.539766 (PMC8022770; doi:10.3389/fsoc.2020.539766)

APPENDIX

Table A1

Top Ten Prize Money Winners in the Alpine and the Nordic Ski World Cup 2017/18 (in CHF)

Rank	Alpine Skiing			
1	Marcel Hirscher	669.681	Mikaela Shiffrin	702.775
2	Henrik Kristoffersen	345.071	Wendy Holdener	273.794
3	Beat Feuz	260.875	Lindsey Vonn	264.430
4	Aksel Lund Svindal	241.000	Viktoria Rebensburg	262.436
5	Kjetil Jansrud	210.250	Sofia Goggia	261.398
6	Vincent Kriechmayr	172.475	Federica Brignone	223.493
7	Thomas Dressen	168.800	Tina Weirather	205.622
8	Alexis Pinturault	142.232	Petra Vlhova	203.955
9	Andre Myhrer	138.259	Frida Hansdotter	148.911
10	Matthias Mayer	108.175	Tessa Worley	139.131
	Nordic Skiing			
1	Johannes Høsflot Klæbo	207.500	Heidi Weng	140.100
2	Alexander Bolshunov	139.625	Ingvild Flugstad Østberg	111.775
3	Sjur Roethe	85.875	Jessica Diggins	109.075
4	Simen Hegstad Krueger	61.050	Marit Bjørgen	94.175
5	Emil Iversen	59.675	Krista Pärmäkoski	79.900
6	Didrik Toenseth	58.775	Charlotte Kalla	65.600
7	Sergey Ustiugov	55.800	Ragnhild Haga	61.000
8	Federico Pellegrino	46.600	Stina Nilsson	53.300
9	Martin Johnsrud Sundby	42.000	Maiken Caspersen Falla	51.250
10	Alex Harvey	35.550	Sadie Bjornsen	41.075

Table A2

Prize Money and World Cup Points by Rank

Rank	Alpine Skiing		Nordic Skiing*	
	World Cup Points	Prize Money (in CHF)	World Cup Points	Prize Money (in CHF)
1	100	45,000	100	15,000
2	80	20,000	80	10,000
3	60	10,000	60	5,000
4	50	7,000	50	3,500
5	45	5,000	45	2,500
6	40	4,000	40	1,500
7	36	3,000	36	1,000
8	32	2,400	32	750
9	29	2,000	29	500
10	26	1,800	26	250
11	24	1,600	24	-
12	22	1,500	22	-
13	20	1,400	20	-
14	18	1,300	18	-
15	16	1,250	16	-
16	15	1,200	15	-
17	14	1,150	14	-
18	13	1,100	13	-
19	12	1,050	12	-
20	11	1,000	11	-
21	10	950	10	-
22	9	900	9	-
23	8	850	8	-
24	7	800	7	-
25	6	750	6	-
26	5	700	5	-
27	4	650	4	-
28	3	600	3	-
29	2	550	2	-
30	1	500	1	-

* In Nordic skiing, prize money varies considerably across the different events. What all the events have in common, however, is that only the first ten finishers receive prize money and that the distribution of the prize purse is the same, irrespective of the amount of money.

Figure A1

The Distribution of World Cup Points and Prize Money in Alpine Skiing

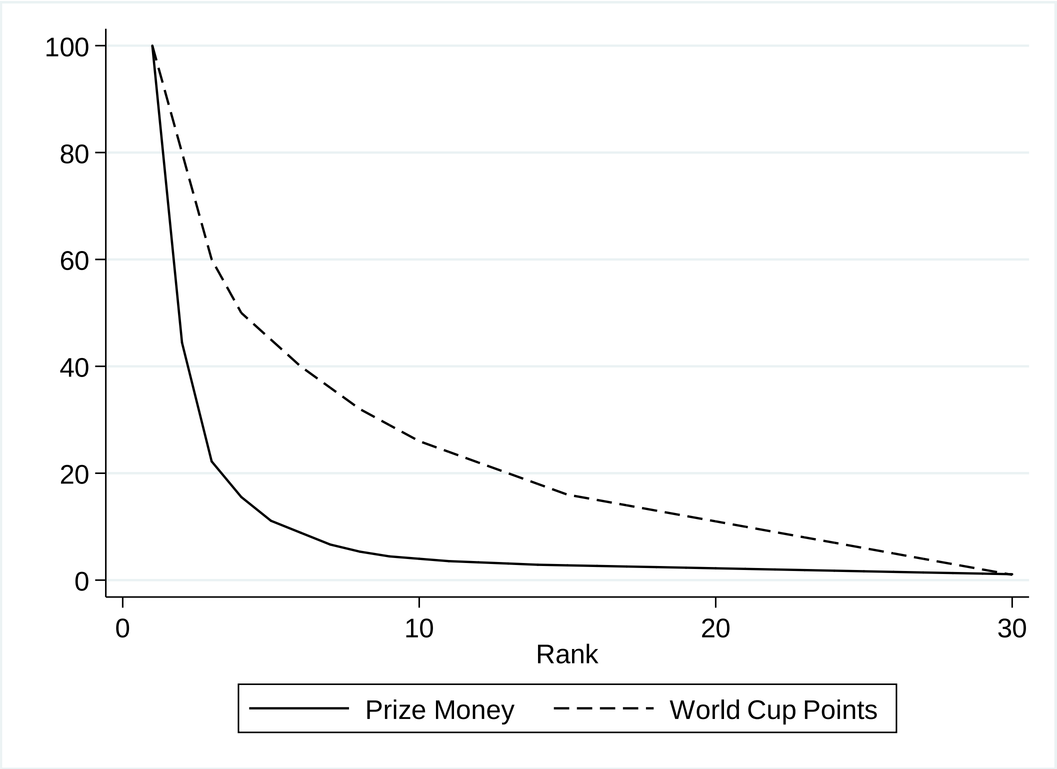

Supplement: Supplementary file 1 [file table1.pdf]
